# Eastern Association for the Surgery of Trauma (EAST) vs Western Trauma Association (WTA): How a Level 1 Trauma Center Splits the Difference in Resuscitative Thoracotomy

**DOI:** 10.7759/cureus.56521

**Published:** 2024-03-20

**Authors:** Moshumi Godbole, Samantha Olafson, Ryan B Cohen, Candace L Ward, Stephanie Sailes, Mia Sharlin, Afshin Parsikia, Benjamin J Moran, Pak Shan P Leung

**Affiliations:** 1 General Surgery, Einstein Medical Center Philadelphia, Philadelphia, USA; 2 General Surgery, Einstein Healthcare Network, Philadelphia, USA; 3 Trauma and Acute Care Surgery, Einstein Healthcare Network, Philadelphia, USA

**Keywords:** ­trauma, resuscitative thoracotomy, blunt thoracic trauma, penetrating thoracic trauma, trauma management

## Abstract

Background

Resuscitative thoracotomy (RT) is performed in severe trauma cases as a final lifesaving effort. Prominent, yet differing, practice management guidelines exist from Eastern Association for the Surgery of Trauma (EAST) and Western Trauma Association (WTA). This study evaluates all RTs performed from 2012 to 2019 at an urban Level 1 trauma center for management guideline indication and subsequent outcomes.

Methods

Our trauma registry was queried to identify RT cases from 2012 to 2019. Data was collected on patient demographics, prehospital presentation, cardiopulmonary resuscitation (CPR) requirements, and resuscitation provided. Survival to the operating room, intensive care unit, and overall were recorded. Information was compared with regard to EAST and WTA criteria.

Results

Eighty-seven patients who underwent RTs were included. WTA guidelines were met in 78/87 (89.7%) of cases, comparatively EAST guidelines were met in every case. Within the EAST criteria, conditional and strong recommendations were met in 70/87 (80.4%) and 17/87 (19.5%) of cases, respectively. In nine cases (10.3%) indications were discordant, each meeting conditional indication by EAST and no indication by WTA. All patients that survived to the operating room (OR), ICU admission, and overall met EAST criteria.

Conclusion

All RTs performed at our Level 1 trauma center met indications provided by EAST criteria. WTA guidelines were not applicable in nine salvaging encounters due to the protracted duration of CPR before proceeding to RT. Furthermore, more patients that survived to OR and ICU admission met EAST guidelines suggesting an improved potential for patient survivability. As increased data is derived, management guidelines will likely be re-established for optimized patient outcomes.

## Introduction

The role for resuscitative thoracotomy (RT) has long been debated in medical literature with many early publications limited by small sample size and unclear indication criteria. Within the last few decades, multiple studies have provided a closer evaluation of RT outcomes and predictors of futility [1-2]. Prominent, yet differing, practice management guidelines have emerged from two major associations; the Eastern Association for the Surgery of Trauma (EAST) and the Western Trauma Association (WTA)[3-4]. Despite guidelines and recommendations, many independent factors contribute towards the decision to initiate RT and there remains variation in provider practice. Data such as age, total injury burden, and comorbidities along with the addition of technological adjuncts have been found to play a role in provider decision making [5]. Concurrently, improved prehospital care and acute resuscitation efforts mean that more patients may be eligible for RT than in years prior. This leads to an increased need for providers to have a clear understanding of the indication and utility of RT. This study evaluates all RTs performed from 2012 to 2019 at an urban Level 1 trauma center for management guideline indication and subsequent outcomes.

This article was previously presented as a meeting abstract at the meeting of the American College of Surgeons in November 2021.

## Materials and methods

The trauma registry at a Level 1 urban trauma center was queried to identify RT cases from 2012 to 2019. Data was collected on patient demographics, prehospital presentation, CPR requirements, and resuscitation measures provided. Outcomes recorded included; survival to the operating room, survival to the intensive care unit, and survival of greater than 24 hours. Exclusion criteria was additionally defined as patient without evidence of thoracotomy, thoracotomy performed in the operating room, and patient’s with mixed traumatic injuries who lacked data regarding the timing of thoracotomy. These criteria were applied prior to performance analysis and the assignment of EAST vs. WTA for each patient. Indication for RT was compared with regard to EAST and WTA criteria.

EAST guidelines describe one strong recommendation and five conditional recommendations. Strong recommendation for RT includes patients who present pulseless but with signs of life after penetrating thoracic injury. Conditional recommendations include; patients who present pulseless without signs of life after penetrating thoracic injury, patients who present pulseless with or without signs of life in extra-thoracic penetrating injury, and patients who present pulseless, but with signs of life after blunt injury. Signs of life include pupillary response, spontaneous ventilation, presence of carotid pulse, measurable or palpable blood pressure, extremity movement, or cardiac electrical activity [3].

WTA indications for RT include patients with blunt traumatic injury with less than 10 minutes of prehospital CPR, patients with penetrating thoracic trauma with less than 15 minutes of CPR, patients with penetrating trauma to the neck or extremity with less than five minutes of prehospital CPR, and patients in profound refractory shock [4]. Table 1 demonstrates a comparison of guidelines utilized by EAST vs WTA. 

**Table 1:** Comparison of guidelines outlined by EAST and WTA which indicate the need for RT SOL = signs of life; RT = Resuscitative thoracotomy.

EAST guidelines	WTA guidelines
Strong: Pulseless with SOL after penetrating thoracic injury	Blunt trauma with <10 minutes of pre-hospital CPR
Conditional: Pulseless without SOL after penetrating thoracic injury Pulseless with SOL after penetrating extra-thoracic injury Pulseless without SOL after penetrating extra-thoracic injury Pulseless with SOL after blunt injury	Penetrating trauma with <15 minutes of pre-hospital CPR or <5 minutes of prehospital CPR with penetrating neck or extremity trauma
	Patients presenting with profound respiratory shock

The dataset was determined, and inclusion criteria were defined as all patients who underwent RT regardless of age, sex, ethnicity, or comorbidity. All patients were adults. There were no cases of prehospital RT and one case where RT was initiated in the operating room. Data determining indication criteria and outcomes were obtained by reviewing the electronic medical record of the emergency medical services-reported data, trauma nurse documentation, code sheet documentation, operative reports, anesthesia reports, and Emergency and Trauma surgery general documentation. A total of 87 patients constituted the sample size. 

Relevant outcomes that were assessed included survival to the operating room, survival to the surgical ICU, and survival of > 24 hours. A survival time of 24 hours was chosen as this is the most critical period in which patients may succumb to fatal traumatic injury and failed resuscitative efforts. In patients who have undergone RT and survived ICU admission, prior studies have shown that as many as 67% will not survive the first 24 hours [6]. This study was reviewed and approved by the Internal Review Board (IRB-2021-586).

## Results

Upon review of the institutional trauma database from 2012 to 2019, a total of 87 patients who underwent RTs were identified and included. WTA guidelines were met in 78/87 (89.7%) of cases. Comparatively, EAST guidelines were met in every case (100%). Within the EAST criteria, conditional and strong recommendations were met in 70/87 (80.4%) and 17/87 (19.5%) of cases, respectively. In the nine cases (10.3%) where indications were discordant, each met conditional indication by EAST and no indication by WTA. All patients that survived to the operating room (OR), ICU admission, and overall met EAST criteria.

An evaluation of outcomes broken down by indication criteria revealed that of those patients who survived in the operating room, 26/26 (100%) met EAST criteria, while 24/26 (92.3%) met WTA criteria. Of the patients that survived to ICU admission 18/18 (100%) met EAST criteria and 17/18 (94%) met WTA criteria. Finally, of the patients who survived beyond 24 hours all 6/6 (100%) met both EAST and WTA criteria. Additionally, of those six who survived, 2/6 (33%) are determined to have had long-term survival defined as greater than 30 days or to discharge, while the remaining 4/6 (67%) were found to expire within 50 days of receiving RT.

Statistical analysis of these outcomes was performed using the student t-test and Mann-Whitney U-test to compare means and medians. Categorical data was analyzed via a Chi-squared test. These outcomes were re-tested with Fisher-exact tests and found to yield comparable results. Tables 2, 3 provide a comparative look at the respective demographics of patients assessed within each guideline, EAST versus WTA, respectively.

**Table 2:** Demographic table (EAST) Statistically significant (P <0.05); EAST = Eastern Association for the Surgery of Trauma.

	Conditional	Strong	p-value
	N=70	N=17	
Age, mean (SD)	32.3(13.5)	31.1 (10.7)	0.73
Age>30	29 (41%)	8 (47%)	0.67
Female	8 (11%)	1 (6%)	0.50
Male	62 (89%)	16 (94%)	0.50
Blunt	9 (13%)	0 (0%)	0.12
Penetrating	61 (87%)	17 (100%)	0.12
ISS, median (IQR)	27.5 (19.5-50.5)	27 (17-75)	0.98

**Table 3:** Demographic table (WTA) Statistically significant (P <0.05); WTA = Western Trauma Association.

	WTA not met	WTA met	p-value
	N=9	N=78	
Age, mean (SD)	35.4(15.0)	31.7 (12.8)	0.42
Age>30	5 (56%)	32 (41%)	0.40
Female	3 (33%)	6 (8%)	0.017
Male	6 (67%)	72 (92%)	0.017
Blunt	4 (44%)	5 (6%)	<0.001
Penetrating	5 (56%)	73 (94%)	<0.001
ISS, median (IQR)	26 (26-33)	28 (19-52.5)	0.76

Table 4 displays the composition of indication criteria met for all patients within the inclusion criteria and the overall breakdown of indication criteria by outcome evaluated. 

**Table 4:** Composition of indication criteria met and breakdown of indication criteria by outcome evaluated RT = Resuscitative thoracotomy; EAST = Eastern Association for the Surgery of Trauma; WTA = Western Trauma Association.

	n	%
WTA guideline	78	89.7
EAST guideline (n=87)		
Conditional	70	80.4
Strong	17	19.5
East and WTA concordance		
EAST and WTA met	78	89.7
Only EAST met	9	10.3
Only WTA met	0	0
Neither met	0	0
Reached operating room	26	29.9
EAST	26	100
WTA	24	92.3
Reached intensive care unit	18	20.7
EAST	18	100
WTA	17	94.4
Overall RT survival	6	6.9
EAST	6	100
WTA	6	100

Tables 5, 6 demonstrate a comparison of outcomes between those patients who met EAST or WTA criteria versus patients who did not meet those criteria, respectively.

**Table 5:** Outcomes (EAST) Statistically significant (P <0.05); OR = Operating room; EAST = Eastern Association for the Surgery of Trauma.

	Conditional	Strong	p-value
	N=70	N=17	
OR	20 (29%)	6 (35%)	0.59
ICU	14 (20%)	4 (24%)	0.75
>24-hour survival	4 (6%)	2 (12%)	0.38

**Table 6:** Outcomes (WTA) Statistically significant (P <0.05); OR = Operating room; WTA = Western Trauma Association.

	WTA not met	WTA met	p-value
	N=9	N=78	
OR	2 (22%)	24 (31%)	0.60
ICU	1 (11%)	17 (22%)	0.45
>24-hour survival	0 (0%)	6 (8%)	0.39

## Discussion

As pre-hospital care and acute traumatic resuscitation continue to improve, it is important to reflect on the current practice of interventions, such as RT, to ensure the best outcomes for patients. The decision to perform RT must be made quickly and despite attempts to provide guidelines, there still exist variability in indication criteria amongst providers.

It has been shown that patients presenting to centers with increased volume of RT have significantly higher odds of survival [7-8]. As a Level 1 trauma center with a high volume of critical trauma patients, it is encouraging that all RTs performed were indicated as per current guidelines. Our overall RT survival rate of 6.9% closely mirrors that of previously published outcomes. For example, Joseph et al. reviewed 25 years of data and ultimately determined an RT survival rate of 7.4% [9]. Unfortunately, this highlights the lack of outcome improvement over two decades since that publication. Furthermore, this study indicates that following the current guidelines alone is not enough to produce dramatic survival benefits [9-10]. 

It is interesting to note that all the RTs performed at our Level 1 trauma center met indications provided by EAST criteria. The discrepancy was ultimately found to be that WTA guidelines were not applicable in nine salvaging encounters due to protracted duration of CPR before performance of RT. One consideration for this finding is that EAST criteria recommendations describe the mechanism of injury and initial clinical presentation, which can allow qualitative assessment of the patient with varied interpretations. Alternatively, the objective component of WTA criteria describing timed cut-offs after cardiopulmonary resuscitative may limit some of the variability for when to perform RT. These guideline differences allow for more clinical cases to fall under EAST criteria than WTA. It is also possible that providers who initially plan to perform RT under WTA guidelines initiate RT after the recommended CPR duration due to unclear length of prehospital CPR performed or other clinical considerations.

Also noted in our data is that more patients that survived to OR and ICU admission met EAST guidelines. It is important to recognize that there likely exists a subset of the patients within the EAST criteria guidelines that are the most likely to benefit from RT and survive to the OR, ICU, and beyond 24 hours.

This study does have limitations, including the discussion of adjunct tools used to aid in decision-making. For example, some institutions have begun to include the use of bedside ultrasound to determine futility and avoid unnecessary RT [11]. This criteria has been used by various trauma surgeons at this particular Level 1 trauma center. Concomitantly, there are patients who historically would have undergone RT but now are managed with resuscitative endovascular balloon occlusion of the aorta, or REBOA placement [12-13]. As more data develops on these adjuncts, it will help to delineate even more appropriate patient populations to undergo RT.

## Conclusions

This study reflected on this single Level 1 trauma center's indication criteria and outcomes for RT over 10 years. Encouraging results indicated that all RTs performed met current practice management guidelines by either EAST, WTA, or both. Interestingly, the data revealed that although all RTs were indicated per current guidelines, outcomes remain particularly morbid and there has been no improvement in survival outcome in comparison to prior studies. As all survivors did meet practice management guidelines, there is more data required to identify how to best optimize practice management guidelines for improved patient outcomes.
